# Perceptions of prognostic risks in chronic kidney disease: a national survey

**DOI:** 10.1186/s40697-015-0088-z

**Published:** 2015-12-20

**Authors:** Helen H. L. Chiu, Navdeep Tangri, Ognjenka Djurdjev, Brendan J. Barrett, Brenda R. Hemmelgarn, François Madore, Claudio Rigatto, Norman Muirhead, Manish M. Sood, Catherine M. Clase, Adeera Levin

**Affiliations:** Nephrology Research, Providence Health Care Research Institute, 4th floor, 1125 Howe Street, Vancouver, BC V6Z 2K8 Canada; BC Provincial Renal Agency, Vancouver, BC Canada; Department of Medicine, Faculty of Medicine, University of Manitoba, Winnipeg, MB Canada; Provincial Health Services Authority, Vancouver, BC Canada; Department of Medicine, Faculty of Medicine, Memorial University of Newfoundland, St. John’s, NL Canada; Division of Nephrology, Foothills Medical Centre, Calgary, AB Canada; Department of Medicine, Faculty of Medicine, Université de Montréal, Montréal, QC Canada; Department of Medicine, Faculty of Medicine, The University of Western Ontario, Hamilton, ON Canada; Ottawa Hospital Research Institute, University of Ottawa, Ottawa, ON Canada; Department of Medicine, Faculty of Medicine, McMaster University, London, ON Canada; Department of Medicine, Faculty of Medicine, The University of British Columbia, Vancouver, BC Canada

**Keywords:** Chronic kidney disease, Risk prediction models, Risk perception, Decision-making, Time frames

## Abstract

**Background:**

Predicting the clinical trajectories of chronic kidney disease (CKD) to discern personalized care remains a complex challenge in nephrology. Understanding the appropriate risk thresholds and time frame associated with predicting risks of key outcomes (kidney failure, cardiovascular (CV) events, and death) is critical in facilitating decision-making. As part of an exploratory research and practice support needs assessment, we aimed to determine the importance of the time frames for predicting key outcomes, and to assess the perceived demand for risk prediction tools among Canadian nephrologists.

**Methods:**

A web-based survey was developed by a pan-Canadian expert panel of practitioners. Upon pre-test for clarity and ease of completion, the final survey was nationally deployed to Canadian nephrologists. Anonymous responses were gathered over a 4-month period. The results were analyzed using descriptive statistics.

**Results:**

One hundred eleven nephrologists responded to our survey. The majority of the respondents described prediction of events over time frames of 1–5 years as being “extremely important” or “very important” to decision-making on a 5-point Likert scale. To plan for arteriovenous fistula referral, the respondents deemed thresholds which would predict probability of kidney failure between >30 and >50 % at 1 year, as useful, while many commented that the rate of progression should be included for decision-making. Over 80 % of the respondents were not satisfied with their current ability to predict the progression to kidney failure, CV events, and death. Most of them indicated that they would value and use validated risk scores for decision-making.

**Conclusions:**

Our national survey of nephrologists shows that the risk prediction for major adverse clinical outcomes is valuable in CKD at multiple time frames and risk thresholds. Further research is required in developing relevant and meaningful risk prediction models for clinical decision-making in patient-centered CKD care.

**Electronic supplementary material:**

The online version of this article (doi:10.1186/s40697-015-0088-z) contains supplementary material, which is available to authorized users.

## ABRÉGÉ

**Contexte:**

Un défi de taille subsiste dans le domaine de la néphrologie. En effet, il demeure difficile de prévoir l’évolution de la néphropathie chronique et donc, d’établir un programme de soin personnalisé pour les patients. Pour ce faire, il serait essentiel de comprendre les seuils de risque ainsi que les marges de temps qui pourraient aider à prévoir l’évolution de la maladie vers les complications inhérentes (l’insuffisance rénale chronique, des événements cardiovasculaires ou la mort). La connaissance de ces paramètres pourrait s’avérer un outil indispensable pour aider les néphrologues à prendre des décisions plus rapidement et plus efficacement quant à la suite du traitement à offrir à leurs patients. Dans le but d’offrir du support à la pratique et de faciliter la prise de décisions cliniques, cette étude exploratoire faite auprès des néphrologues canadiens visait à évaluer l’importance d’établir des cadres temporels et des seuils de risque permettant de prévoir l’évolution défavorable de la néphropathie chronique vers ses principales complications.

**Méthode:**

Un groupe d’experts provenant de tous les coins du pays a mis au point un sondage sur le web pour questionner directement les néphrologues. Une version pilote du test a d’abord été distribuée à un groupe restreint pour en vérifier la clarté et faire en sorte de le rendre le plus convivial possible pour les répondants. La version définitive été envoyée par courriel à des néphrologues pratiquant à travers le Canada. Les réponses au questionnaire ont été compilées en préservant l’anonymat des répondants, sur une période de quatre mois, et ont été analysées par statistique descriptive.

**Résultats:**

Un total de cent-onze néphrologues ont répondu au sondage, et sur une échelle de Likert en cinq points, la majorité d’entre eux a qualifié d’« extrêmement importante » ou de « très importante » pour faciliter la prise de décision, la possibilité de prévoir les événements défavorables sur une période de un à cinq ans. Dans le cas précis de la pose d’une fistule artérioveineuse, une bonne proportion de répondants a mentionné qu’ils la recommanderaient à leurs patients s’ils pouvaient estimer le risque de développer une insuffisance rénale chronique à plus de 50 % à l’intérieur d’une période d’un an, alors que d’autres ont mentionné qu’ils le feraient si ce risque se situait à plus de 30 % pour la même période. Tous ont mentionné qu’ils trouveraient utile d’avoir la capacité de prévoir une telle issue, et plusieurs ont ajouté que le rythme de progression de la maladie devrait être considéré dans la prise de décision. En tout, plus de 80 % des répondants se sont dits insatisfaits de leur capacité actuelle à prévoir la progression de la maladie vers l’insuffisance rénale chronique, des complications d’ordre cardiovasculaires ou la mort. Enfin, la plupart d’entre eux ont indiqué qu’ils jugeraient utile la création d’une charte validée de seuils de risques permettant de prédire l’évolution défavorable de la maladie et qu’ils seraient enclins à l’utiliser dans leurs prises de décisions.

**Conclusion:**

Les résultats de ce sondage national effectué auprès de néphrologues-praticiens montrent que la capacité de prévoir, dans une gamme de périodes de référence donnée, les risques de résultats cliniques défavorables chez les patients atteints de néphropathie chronique serait grandement utile pour la prise de décisions quant au choix du traitement. Des recherches plus approfondies sont requises en vue de développer des modèles pertinents et concrets de prévision des risques d’évolution défavorable de la maladie, dans le but de faciliter la prise de décisions cliniques et d’offrir aux patients des soins axés sur leurs besoins particuliers.

## What was known before

Much effort has been spent and resources expended on the development of equations and decision support tools for predicting the risks of key outcomes, such as end-stage renal disease, cardiovascular events, and death, for people with advanced CKD. However, the need for risk prediction tools has yet to be explored.

## What this adds

The perception and attitudes of nephrologists about the prediction of key clinical outcomes in advanced CKD with validated risk prediction tools were explored in a national survey. Our findings show that the risk prediction for major adverse clinical outcomes is valuable in CKD at multiple time frames and risk thresholds, prompting further research and knowledge translation in the development and adaptation of risk prediction tools to guide clinical decision-making.

## Background

Chronic kidney disease (CKD) is a global health problem. In Canada, CKD affects about 3 million adults [1], over 10 % of the Canadian population, and a substantial proportion progress to end-stage renal disease (ESRD). Patients with CKD are at increased risks for kidney failure, cardiovascular (CV) events, and death [2–6]. Both patients and care providers face a constellation of decision-making challenges, specifically in regard to the anticipation of potential needs for renal replacement therapy (transplant or dialysis planning), intensive treatment for CV risk reduction or planning for conservative care. There have been limited attempts to understand the impact of using prediction tools to guide the decision-making for the patients and clinicians.

The absence of evidence-based information guiding both the patients and providers may result in delay of decision-making and leads to emergency hemodialysis starts, or starting dialysis with a catheter instead of a fistula, both of which have been shown to increase the patients’ risk for serious adverse outcomes [7–9]. Current best evidence would suggest that commencing dialysis with an arteriovenous fistula (AVF) results in the best outcomes for patients and remains the most preferred vascular access due to better long-term survival of the access, ease of maintaining patency, lower complication rates, and mortality compared with other options [9–14]. Nevertheless, in Canada, fewer than 16 % of patients commence hemodialysis with an AVF [15]. Furthermore, adequate knowledge of risk prediction may allow appropriate end-of-life planning with timely advance care planning and integrated palliative approach to care that enables better quality of life nearing the end of life for the patients with CKD [16]. There is a need to better understand the factors that contribute to the challenge of improving decision support, so as to optimize outcomes for patients and the health care system.

Previous studies have been conducted to identify available risk prediction models for important outcomes in CKD [17]. These studies have shown that validated risk prediction models exist for predicting kidney failure, but development and validation efforts are needed to predict CV events and mortality. While such efforts are important in understanding and improving the “performance” of various risk prediction tools, a thorough assessment of the practice support needs for risk prediction models in CKD has not yet been conducted.

We conducted a national survey to determine the importance of specific time frames for prediction of key outcomes from the perspective of nephrologists. Time frames were considered in the context of the estimated length of time that is considered when making clinical care decision. In addition, we were interested in exploring what specific thresholds of risk would lead clinicians to change treatment plans with the patients. This information would be helpful to guide knowledge translation strategies to enhance the uptake of tools that help clinicians to predict these risks.

## Methods

A survey for nephrologists was developed by an expert panel composed of Canadian nephrologists, methodologists, researchers and administrators knowledgeable in risk prediction modeling, decision support, and knowledge translation in renal care. During a face-to-face meeting, the expert panel defined the duration for short- and long-term time frames along with other key issues around tools that aim to predict risk of outcomes important to both clinicians and patients. Targeting a representative group of practicing nephrologists in Canada as our potential respondents, a series of questions were designed and vetted to determine a set for testing. A pilot test of the survey was done by a small group of nephrologists to ensure clarity of the questions and ease of completion. Minor modifications yielded the final survey consisting of nine questions (Additional file 1).

The domains of interest were those of (a) time frames and (b) thresholds of certainty of prediction, in terms of levels of risk and estimated glomerular filtration rate (eGFR), which would inform decision-making for key events, requiring actions or interventions by clinicians or patients. Specifically, we asked the respondents to rate the importance of (1) their ability to predict the risk of kidney failure (requiring dialysis or transplantation) for their individual patients with eGFR 15–45 ml/min/1.73 m^2^ over 1, 3, 5, 10, and 15 years; (2) clinical thresholds for vascular access planning; and (3) their experiences with regards to existing risk prediction tools for clinical decision-making with the patients (e.g., dialysis and transplant planning, discussion of cardiovascular (CV) risk reduction strategies (e.g., lipid-lowering), and end-of-life planning). The nephrologists were also asked if they would use risk prediction tools, if they were readily available and validated, for discerning disease management options with the patients, and what else they would need to facilitate decision-making. Responses to these questions were in 5-point Likert scale or multiple-choice format. “Other, please specify” option with open text responses was included where appropriate. All survey responses were anonymous.

The web-based exploratory survey was progressively deployed nationally by snowballing approach [18]. In this approach, an invitation e-mail with the website address hosting the web-based survey were sent to the e-mail lists of provincial renal networks and the Canadian KidNey Knowledge TraNslation and Generation NeTwork (CANN-NET) [19]. The recipients were encouraged to forward the invitation to their colleagues. The choice of the sampling method is to enable rapid response and broad reach to nephrologists who are outside of a specialized professional community and may not otherwise have been reached. Responses between December 2012 and April 2013 were captured in the analysis. Providence Health Care Research Ethics Board screened and acknowledged that this work qualifies for an exemption of institutional ethics review under the Tri-Council Policy Statement on Ethical Research Involving Humans.

**Fig. 1:**
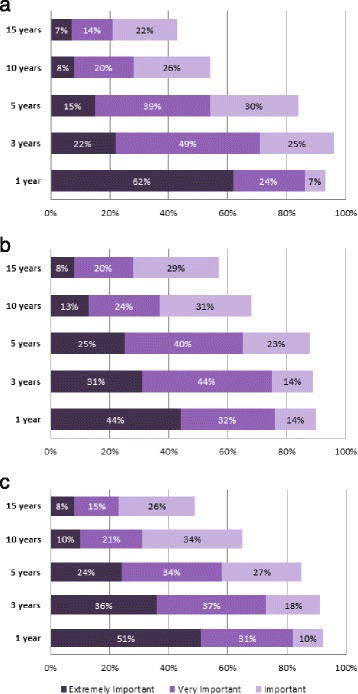
Importance for nephrologists of having the ability to predict risk of **a** kidney failure for individual patients, **b** CV events, and **c** death over specific time frames

Electronic survey data were downloaded, compiled, and analyzed in Microsoft Excel (version 2010; Microsoft Corporation, Redmond, WA). With the snowball sampling, it was impossible to calculate the actual response rate of the survey; thus, the survey penetration was estimated as the proportion of responses to the total number of nephrologists across the country at the time of the survey. Descriptive statistics were generated to summarize individual responses to each question and presented in bar charts or in text. For questions with responses on a 5-point Likert scale, the number of responses for each option was presented as a percentage to the total number of responses of each question. For the questions around importance of time frames (Fig. 1), “extremely important”, “very important”, and “important” were deemed as positive responses.

## Results

One hundred and eleven nephrologists across Canada responded to the surveys over the 4-month period, representing 20 % of practicing nephrologists in Canada. To determine how important the various time frames (1, 3, 5, 10, and 15 years) were perceived by nephrologists for predicting various key clinical outcomes, respondents were asked to rate the importance of predicting the risk of each outcome: kidney failure, CV events, and death in their individual patients with eGFR 15–45 ml/min/1.73 m^2^. Over 80 % of respondents felt that the time frames of 1, 3, and 5 years were most relevant in predicting risk of each outcome: kidney failure, CV events, and death in their patients (Fig. 1a–c). In particular, a higher proportion of respondents rated as extremely important the ability to predict the risk of kidney failure (62 %) as opposed to the risk of CV events (44 %) or death (51 %). The importance of rating these risks dropped for the longer time frames: <70 % of respondents rated the time frames of 10 or 15 years as important. A small subset of respondents expressed that their abilities to predict the risk of kidney failure (1 % of respondents), CV events (4 %), or death (2 %) did not matter to them or that they did not think of risks in the described manner.

**Fig. 2:**
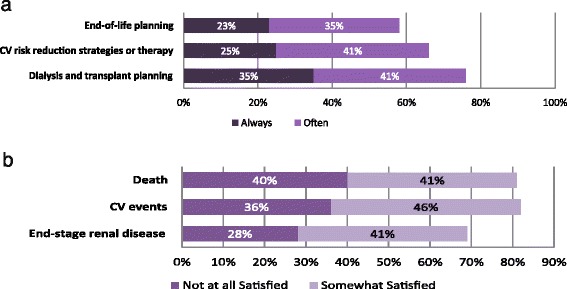
Predicting risks in CKD management for nephrologists. **a** Frequency that nephrologists would use a validated risk score for predicting specific outcomes in order to discuss options in CKD management with their patients. **b** Levels of satisfaction among nephrologists for their current ability to predict specific outcomes in their patients

To assess the needs for the development of risk modeling and tools for informing the clinical management of CKD, we asked our respondents about the perceived utility and value of having validated risk scores for predicting the key clinical outcomes. As shown in Fig. 2a, out of the 5-point scale, most of our survey respondents indicated that they would “always” or “often” use a validated risk score for predicting specific outcomes in order to initiate dialysis and transplant planning (76 % of respondents selected one of those choices), CV risk reduction strategies (66 %), or end-of-life planning (58 %) with their patients. Since uptake among physicians for a validated risk prediction score in making decisions about clinical management may be dependent on the satisfaction levels they might have with the current prediction methods, we asked about satisfaction with their current certainty to predict specific outcomes for their patients with eGFR 15–45 ml/min/1.73 m^2^. The majority of the respondents were not satisfied with their ability to predict the progression to kidney failure, CV events, and death (Fig. 2b). Specifically, the ability of the clinicians to predict CV events and death is most dissatisfying for them with 82 and 81 %, respectively, rated “not at all satisfied” or “somewhat satisfied”.

**Fig. 3:**
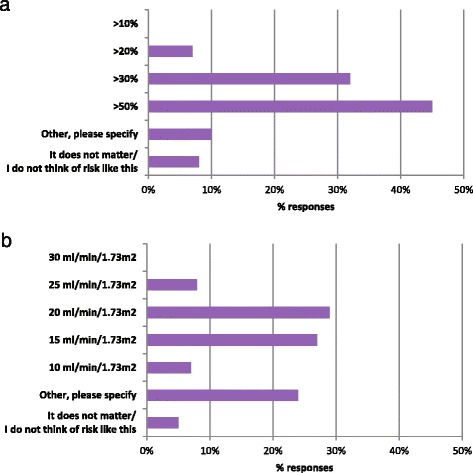
Acceptable thresholds for AVF referral. **a** 1-year risk of kidney failure for AVF referral **b** eGFR thresholds for AVF referral

In assessing factors relevant to vascular access planning for kidney failure, we asked nephrologists what 1-year risk threshold for kidney failure would prompt them to refer for AVF creation in their patients who had chosen hemodialysis as a treatment option. In response, 45 % of respondents would refer their patients for AVF if the risk of kidney failure were >50 %, while 32 and 7 % of respondents would refer at risk thresholds of >30 and >20 %, respectively (Fig. 3a). The respondents who chose “other” remarked they would only refer if the risk of kidney failure is much higher and with consideration of local resources available, i.e., access to surgery and transplantation. When asked at what eGFR they would refer their patients for AVF, the majority of the responses were split among 15 ml/min/1.73 m^2^ (27 % of respondents), 20 ml/min/1.73 m^2^ (29 %), and “other” (24 %) (Fig. 3b). As much as 73 % of respondents who chose “other” commented that consideration of the rate of progression or progressive decline in GFR would also be necessary for the decision-making.

Most respondents (39 % “yes, maybe”; 61 % “yes, definitely”) indicated that they would use validated risk scores accurate to predict specific outcomes, if they were available. They would more likely use risk scores (29 % “yes, maybe”; 71 % “yes, definitely”) if the clinical management (i.e., timing of education about ESRD management, planning for access, referral for transplant, and commencement of medications) of their individual patients would be altered to improve the outcome.

## Discussion

This is the first study, to our knowledge, to assess nephrologists’ perceptions and attitudes to predicting key outcomes related to validated risk and prediction tools. Our findings describe how nephrologists perceive the importance of risk prediction over different time frames and identify specific considerations in the context of clinical decision-making with patients with CKD. Most predictive models at present are based on observational cohorts with average follow-up periods of less than 10 years, [17, 20–22] which may explain our nephrologist respondents’ focus on the shorter time frames. Nephrologists appear to feel that remote events are harder to predict for individual patients and perhaps less relevant for their immediate decision making on care plans. This is interesting given published observational studies in CKD prognosis with long-time frames, including lifetime risk [23–25]. Outside of nephrology, there are many examples of valuable cohorts followed over extended time periods e.g., Framingham, the Nurses’ Health Study, the Cardiovascular Health Study [26–28]. These studies describe 10- and 20-year risks of important events.

The survey identified a need for validated risk scores to guide the clinical management of CKD. Satisfaction levels with regards to current methods for predicting the key clinical outcomes were very low for the majority of respondents, particularly for predicting CV events and death. The findings correspond to the poor availability of these validated tools other than those for predicting renal failure in patients with CKD [17]. With regard to the prediction of CV events, the Framingham score, widely used to identify CV risk in people without advanced kidney disease [26, 29, 30] is known to have a poor calibration and discrimination in patients with CKD [30]. Furthermore, CKD patients are under-treated for conventional cardiovascular disease (CVD) risk factors, and, although a large number of post-hoc analyses have examined the effect of CKD on CVD, patients with advanced stages of CKD have not been included in most CVD clinical trials [31–33]. The respondents strongly supported the utility of improved risk scores with potential for impact on the clinical management of CKD in their individual patients. There appears to be an urgent need to develop and validate prediction tools for CV events and death as well as clinical trials for treatment strategies in CKD populations. Moreover, based on these results, uptake of risk scores for the key clinical outcomes in CKD is likely to be favorable if relevant research is developed to concurrently test the actual utility and effectiveness of these risk scores for improving patient care in practice.

In terms of decision-making regarding vascular access planning for possible dialysis, the respondents deemed >30 and >50 % of 1-year risk of kidney failure most appropriate to inform referral. If choosing a specific level of eGFR for referral, both the 15 ml/min/1.73 m^2^ and 20 ml/min/1.73m^2^ eGFR levels were deemed equally acceptable to trigger AVF referral. The results are concordant with the Canadian Society of Nephrology guideline which recommends AVF referral at eGFR of 15–20 ml/min/1.73m^2^ in the setting of progressive kidney disease [10]. A vignette-based survey has shown distinct variability of Canadian and American nephrologists in criteria used to guide vascular access referral, but the specific thresholds of risk by which the nephrologists would make such decision were not explored [34]. Since individual patients experience varying trajectories of CKD, often complicated with the trajectories of co-morbidities, [23, 35, 36] the rate of progression, as suggested by many respondents, and additional factors should be considered in determining optimal timing for AVF referral. How vascular access information is presented and the timing of communicating the information to the patients also influenced their decision to accept or refuse an AVF [37].

While this survey focused on exploring the perceptions of the nephrologists, it is of note that patients and their clinicians often view illness trajectories and key transitions quite distinctly from each other [38]. Some differences in perception also stem from the presence of information asymmetry in the principal-agent relationship, in which the patients (principals) often lack the clinical and technical knowledge of the providers. Physicians are generally better informed of the risks and aspects of the diseases than the patients [39]. While communication of prognosis is generally lacking in practice, the vast majority of patients with CKD feel that it is critically important to be informed about their prognosis [40]. Thus, future studies for predicting the risks of key outcomes will benefit from understanding the preference of the patient and their perceptions of the time frames and risk threshold. Such investigations may illuminate communication in shared decision-making and thus better inform a truly patient-centered approach to CKD care.

Our survey findings should be interpreted in light of some limitations. While an estimate of 20 % coverage of nephrologists in Canada for this type of survey is expected, the generalizability to all nephrologists in Canada or to other countries is fairly limited, and the results may not be representative of all nephrologists. With the nature of the sampling technique and without being able to determine the number of pediatric nephrologists, to which this survey is not applicable, the estimated response rate using the total number of practicing nephrologists is an under-estimation. Furthermore, our coverage was similar to the response rate of other studies using survey instruments [41–43]. We did not ask respondents to specify their age or other demographic information about the respondents and so was not able to ascertain the relationship between individual factors and perceptions. The surveys aimed to understand which “thresholds” of risk would trigger action, but did not ask if the clinicians were actually using prediction scores in this manner. The exploratory survey was not supplemented by focus groups or interviews, which would add depth to our results, but the results of which will guide the planning of these activities currently underway.

## Conclusions

Our national survey of nephrologists shows that the risk prediction for major adverse clinical outcomes is valuable in CKD at multiple time frames and risk thresholds. The nephrologists studied tended to value shorter term predictions more than longer term, perhaps because current evidence primarily relies on observational studies of short time frames. Ongoing research should focus on the development of better prediction tools, but also determine how patients and clinicians can use these prediction models in decision-making activities. Understanding how patients and clinicians perceive prognosis can help collectively to develop appropriate clinical management strategies and care, and develop tools to inform that care.

## Additional file

Additional file 1:
**Survey on the perceptions of prognostic risks in CKD to Canadian nephrologists.** (PDF 82 kb)

## Abbreviations

AVF: arteriovenous fistula
CANN-NET: Canadian Kidney Knowledge Translation and Generation Network
CKD: chronic kidney disease
CV: cardiovascular
CVD: cardiovascular disease
ESRD: end-stage renal disease
