# Supplementary material for: Perceptions of prognostic risks in chronic kidney disease: a national survey
Source: Can J Kidney Health Dis. 2015 Dec 20;2:53. doi: 10.1186/s40697-015-0088-z (PMC4684914; doi:10.1186/s40697-015-0088-z)
Supplement: Additional file 1: — Survey on the perceptions of prognostic risks in CKD to Canadian nephrologists. (PDF 82 kb) [file 40697_2015_88_MOESM1_ESM.pdf]

Question 1. Is it important for you to be able to predict the risk of kidney failure requiring dialysis or transplantation for your individual patients with eGFR 15-45 ml/min/1.73m2 over the next…

	Not at all Important	Somewhat Important	Important	Very Important	Extremely Important
1 year	○	○	○	○	○
3 years	○	○	○	○	○
5 years	○	○	○	○	○
10 years	○	○	○	○	○
15 years	○	○	○	○	○

☐ It does not matter/ I do not think of risk of kidney failure like this

Question 2. Is it important for you to be able to predict the risk of cardiovascular (CV) events (i.e. ischemic heart disease, congestive heart failure) for your individual patients with eGFR 15-45 ml/min/1.73m2 over the next…

	Not at all Important	Somewhat Important	Important	Very Important	Extremely Important
1 year	○	○	○	○	○
3 years	○	○	○	○	○
5 years	○	○	○	○	○
10 years	○	○	○	○	○
15 years	○	○	○	○	○

☐ It does not matter/ I do not think of risk of CV events like this

Question 3. Is it important for you to be able to predict the risk of <u>death</u> for your individual patients with eGFR 15-45 ml/min/1.73m2 over the next…

	Not at all Important	Somewhat Important	Important	Very Important	Extremely Important
1 year	○	○	○	○	○
3 years	○	○	○	○	○
5 years	○	○	○	○	○
10 years	○	○	○	○	○
15 years	○	○	○	○	○

☐ It does not matter/ I do not think of risk of death like this

Question 4. Would you use a validated risk score specific for predicting outcomes with your patients with kidney disease for the following:

	Never	Rarely	Sometimes	Often	Always
Dialysis and transplant planning	○	○	○	○	○
CV risk reduction strategies or therapy (e.g. lipid lowering)	○	○	○	○	○
End of life planning	○	○	○	○	○

Question 5. If your ability to accurately predict specific outcomes was demonstrated to be improved, would you use the risk score?

○ No

○ Yes, may be

○ Yes, definitely

Question 6. If your clinical management of individual patients would be altered (i.e. timing of education about end-stage renal disease management, planning for vascular or PD access, referral for transplant, commencement of medications), would you use the risk score?

- ○ No
- ○ Yes, may be
- ○ Yes, definitely

Question 7. At what 1-year risk for kidney failure requiring dialysis or transplantation would you refer your patients for arteriovenous fistula (AVF), if they have chosen hemodialysis?

- ○ >10%
- ○ >20%
- ○ >30%
- ○ >50%
- ○ Other, please specify ___________________
- ○ It does not matter/ I do not think of risk of kidney failure like this

Question 8. At what estimated GFR would you refer your patients for AVF, if they have chosen hemodialysis?

- ○ 30 ml/min/1.73m2
- ○ 25 ml/min/1.73m2
- ○ 20 ml/min/1.73m2
- ○ 15 ml/min/1.73m2
- ○ 10 ml/min/1.73m2
- ○ Other, please specify ___________________
- ○ It does not matter/ I do not think of risk of kidney failure like this

Question 9. Are you satisfied with your current ability, based on clinical judgment and available risk prediction instruments, to predict the following events in individual patients?

	Not at all Satisfied	Somewhat Satisfied	Satisfied	Very Satisfied	Extremely Satisfied
End-stage renal disease	○	○	○	○	○
CV events	○	○	○	○	○
Death	○	○	○	○	○
